# A daily gap-free normalized difference vegetation index dataset from 1981 to 2023 in China

**DOI:** 10.1038/s41597-024-03364-3

**Published:** 2024-05-22

**Authors:** Huiwen Li, Yue Cao, Jingfeng Xiao, Zuoqiang Yuan, Zhanqing Hao, Xiaoyong Bai, Yiping Wu, Yu Liu

**Affiliations:** 1 Shaanxi Key Laboratory of Qinling Ecological Intelligent Monitoring and Protection, School of Ecology and Environment, Northwestern Polytechnical University, Xi’an, Shaanxi Province 710129 China; 2 Technology Innovation Center for Natural Ecosystem Carbon Sink, Ministry of Natural Resources, Kunming, Yunnan Province 650111 China; 3 Xi’an Institute for Innovative Earth Environment Research, Xi’an, Shaanxi Province 710061 China; 4 Earth Systems Research Center, Institute for the Study of Earth, Oceans, and Space, University of New Hampshire, Durham, NH 03824 USA; 5 State Key Laboratory of Environmental Geochemistry, Institute of Geochemistry, Chinese Academy of Sciences, Guiyang, Guizhou Province 550081 China; 6 Department of Earth & Environmental Science, Xi’an Jiaotong University, Xi’an, Shaanxi Province 710049 China

**Keywords:** Ecosystem ecology, Forest ecology

## Abstract

Long-term, daily, and gap-free Normalized Difference Vegetation Index (NDVI) is of great significance for a better Earth system observation. However, gaps and contamination are quite severe in current daily NDVI datasets. This study developed a daily 0.05° gap-free NDVI dataset from 1981–2023 in China by combining valid data identification and spatiotemporal sequence gap-filling techniques based on the National Oceanic and Atmospheric Administration daily NDVI dataset. The generated NDVI in more than 99.91% of the study area showed an absolute percent bias (|PB|) smaller than 1% compared with the original valid data, with an overall *R*^2^ and root mean square error (RMSE) of 0.79 and 0.05, respectively. *PB* and RMSE between our dataset and the MODIS daily gap-filled NDVI dataset (MCD19A3CMG) during 2000 to 2023 are 7.54% and 0.1, respectively. *PB* between our dataset and three monthly NDVI datasets (i.e., GIMMS3g, MODIS MOD13C2, and SPOT/PROBA) are only −5.79%, 4.82%, and 2.66%, respectively. To the best of our knowledge, this is the first long-term daily gap-free NDVI in China by far.

## Background & Summary

Vegetation indices (VIs) are pivotal tools for assessing vegetation conditions. Among all VIs, Normalized Difference Vegetation Index (NDVI) is one of the most popular indices due to its high reliability and easy accessibility^1^. This index has been widely used in many fields, such as identification of vegetation phenology^2^, assessment of biomass production^3^, estimation of crop yield^4^, evaluation of ecosystem stability^5^, simulation of vegetation carbon sequestration^6^, and fields related to ecosystem status and functions^7–15^. In addition, many new VIs use NDVI as the basic data to optimize the estimation of some key parameters of the terrestrial biosphere (e.g., leaf area index, gross primary productivity, and sun-induced chlorophyll fluorescence) through nonlinear generalization of NDVI, such as the near-infrared reflectance of terrestrial vegetation (NIRV)^16^ and the kernel NDVI (kNDVI)^17^. Therefore, obtaining long term, high temporal resolution (e.g., daily scale), and high accurate NDVI is of great significance for better monitoring the multiple properties of vegetation.

To the best of our knowledge, there are currently two datasets offering daily continuous NDVI: one is the Moderate Resolution Imaging Spectroradiometer (MODIS) MCD19A3CMG v6.1, and the other is the National Oceanic and Atmospheric Administration (NOAA) Climate Data Record (CDR) program. MCD19A3CMG is a global Terra + Aqua combined MODIS Version 6.1 Multi-Angle Implementation of Atmospheric Correction (MAIAC) data dataset, which provides a global daily gap-free NDVI dataset at 0.05° spatial resolution in a global Climate Modeling Grid (CMG) from February 24, 2000 to the present^18^. Apart from spatial gaps in the NDVI maps on the first few days, the MCD19A3CMG v6.1 NDVI maps are generally complete at other time points. However, its limitation is that data is only available after February 24, 2000. The NOAA CDR program generated a long-term global gridded daily NDVI dataset spanning from 1981 to the present by integrating AVHRR daily surface reflectance from eight NOAA polar orbiting satellites (NOAA-7, −9, −11, −14, −16, −17, −18, and −19), VIIRS daily surface reflectance from the Suomi National Polar-Orbiting Partnership (SNPP) satellite platform and NASA-NOAA Joint Polar Satellite System (JPSS-1 or NOAA-20), and other ancillary data (e.g., digital elevation model, surface pressure, precipitable water, water vapor, and ozone) at 0.05° spatial resolution^19–21^. However, the NOAA CDR NDVI dataset did not fully address the issue of spatial gaps caused by the interference of noise sources. Gaps and noise data are still severe in the NOAA CDR NDVI dataset.

Certainly, a series of methods have been developed to rectify, fill, and reconstruct the contaminated remote sensing NDVI data^22^. These methods can be broadly categorized into three categories: temporal-based methods (i.e., temporal interpolation-replacement methods, temporal filtering models, temporal function-fitting models, and temporal deep learning models), frequency-based methods, and hybrid methods. These methods have their respective advantages and disadvantages, as well as assumptions and application scopes^23^. Temporal interpolation-replacement methods may yield subpar outcomes when confronted with extensive data gaps due to their sensitivity to data quality and sample size^24^. Temporal filtering models are mainly used to smooth NDVI curves, relying on the sizes of sliding windows and filtering functions. The size of the sliding window and the filtering function are predicated on prior knowledge^25–27^, and these methods are particularly susceptible to data plunges or surges^28^. It is hard to obtain reliable parameters required for temporal function-fitting models when there is much noise in the original data^29^, which poses challenges for the application of these methods on a large scale. Temporal deep learning models can effectively mitigate noise in most cases, except when there are long temporal gaps that may reduce reconstruction accuracy^28^. Frequency-based methods require a considerable number of parameters, and perform poorly with irregular time series data^30^. Some models may exhibit suboptimal performance when there are high-frequency variations^31^. Hybrid methods consider both temporal continuity and spatial similarity for data denoising. These methods assume that the vegetation phenology in different areas of the same vegetation type in the influence window is the same, and there are discontinuous clouds in space. Therefore, adjacent pixels can be used to reduce the contamination of the target pixel. Hybrid methods can better utilize information in both spatial and temporal domains, but their reliability is influenced by window size, accuracy of the vegetation type, and cloud range^32^. In summary, the aforementioned methods aim to fill gaps and pay little attention to identifying valid data. However, these gap-filling methods require reliable original valid data in terms of both spatial coverage and temporal density. They can hardly reconstruct reliable results if data is missing or unavailable due to cloud or other noise on a large scale^28^. Therefore, there is an urgent need to develop a method that can identify valid data and fill gaps simultaneously, in order to reconstruct long-term, daily, and gap-free NDVI data.

This study aims to establish a framework to reconstruct daily gap-free NDVI data by combining valid data identification and spatiotemporal sequence gap-filling techniques based on the NOAA CDR NDVI. We applied the aforementioned framework to China and, through a systematic process of validation and assessment, reconstructed continuous spatiotemporal maps of daily NDVI spanning from June 1981 to the May 2023 in China.

## Methods

### NDVI reconstruction framework

**Fig. 1:**
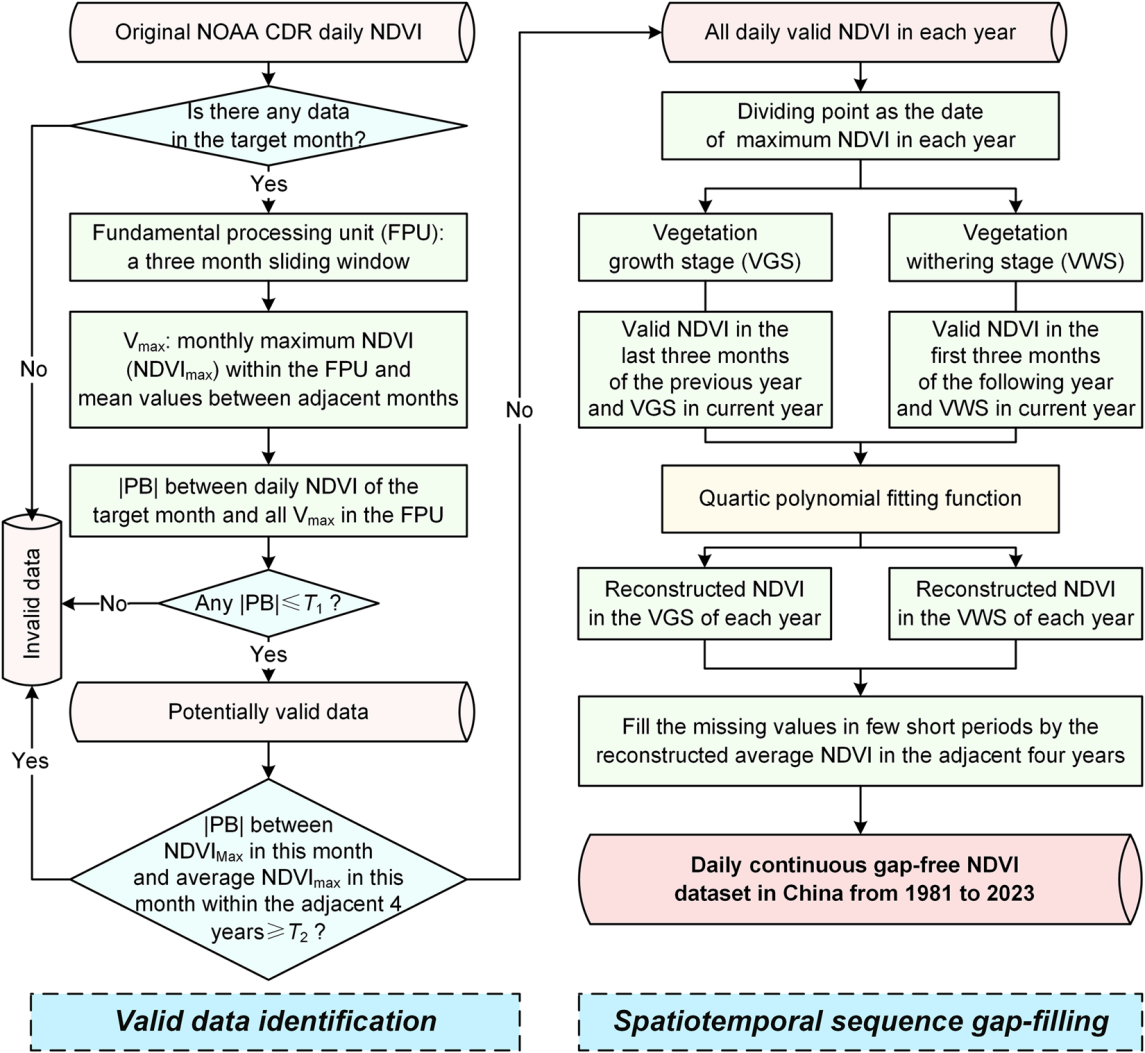
The NDVI reconstruction framework.

Reconstructing a daily continuously valid NDVI dataset with no gaps requires addressing two issues: the identification of valid data and the filling of invalid data. To tackle these challenges, we conceived a reconstruction framework of daily NDVI that combines a daily-scale valid data identification algorithm and spatiotemporal sequence gap-filling technique (Fig. 1). To minimize the cumulative impact of errors introduced by different data sources and enhance the practicality of the reconstruction framework, our approach relies solely on the original NDVI CDR daily NDVI, without incorporating additional auxiliary data.

We constructed a sliding window as the fundamental processing unit (FPU). NDVI in the early segment (e.g., the first 10 days) of the target month may be closer to the month-end level of the preceding month. On the contrary, NDVI in the later stage (e.g., the last 10 days) of the target month may tend to be more similar to the level at the start of the following month. Meanwhile, NDVI of the middle of the target month may be closer to the maximum value of this month. Therefore, this study sets the FPU to a duration of three months. For each pixel, we hunt out the maximum NDVI (NDVI_max_) of each month within the FPU and calculated their mean values between the target month and two adjacent months (five numbers in each FPU, here recorded as V_max_). Then, we calculate the percent bias (*PB*) between daily NDVI of the target month and V_max_ in the corresponding FPU. If the absolute *PB* (|PB|) between the daily NDVI and any of the V_max_ do not exceed a specific threshold *T*_1_, NDVI in this day can be deemed as potentially valid data, otherwise, it was identified as invalid data. If the pixel lacks data within the target month (e.g., short gap periods during satellite replacement)^21^ or the |PB| between NDVI_max_ in the target month and mean value of NDVI_max_ in the same month within the adjacent four years exceed a specific threshold *T*_2_, data in the target month is identified as invalid data. The threshold *T*_1_ is a critical parameter, serving as the key criterion for delineating valid data. If this threshold is too large, there is a risk of misclassifying noise data as valid data. Conversely, if it is too small, it may lead to the omission of some valid data, especially during periods that vegetation grows (e.g., May) or wilts (e.g., October) rapidly. The threshold *T*_2_ is used to further filter out the situation that there is no valid data throughout the entire month due to contamination. If this threshold is too large, noise data may be misclassified as valid records. On the contrary, if the threshold is too small, some valid data may be ignored due to substantially interannual variations in NDVI during some periods. We used the GIMMS3g monthly maximum NDVI during 1982 through 2015 to calculate the two thresholds. The national average |PB| of adjacent months for all months is about 20.39% (Supplementary Figure S1a). Hence, we set the threshold *T*_1_ as 20%. The national average value of the maximum |PB| of all months is about 39.26% (Supplementary Figure S1b). We set the threshold *T*_2_ as 39%. This valid data identification approach fully considers the trend of data changes within the time window, and determines the availability of the data by setting |PB| thresholds. This strategy helps to reduce the impact of possible outliers while retaining sensitivity to vegetation changes. The above identification algorithm can eliminate invalid and missing values in the original NOAA CDR daily NDVI dataset, and provide important baseline data for the next step of data reconstruction.

Considering the asymmetric distribution of the vegetation greenness curves during growth and withering phases^33^, this study utilized the segmented fitting function for the reconstruction of daily NDVI. For each pixel, based on the extracted daily valid NDVI of each year, we identified the date of the maximum value for that year and determined it as a dividing point to categorize the dates into vegetation growth and withering stages. We stacked the valid NDVI in the last three months of the previous year and valid NDVI in the current year’s growth stage. Similarly, for the withering stage, we combined the valid NDVI in the first three months of the following year and valid NDVI in the current year’s withering stages. These two valid NDVI collections constitute the foundational data segments for reconstructing the daily NDVI during the growth and withering stages of the target year. We used the quartic polynomial fitting function to construct daily NDVI fitting functions for the growth and withering stages of each year at the pixel scale based on the valid NDVI records, respectively. Then, these functions were separately applied for the reconstruction of daily NDVI in these two segments. Finally, a continuous daily gap-free NDVI dataset from 1981 to 2023 was constructed by iterating through all dates for each pixel. Considering the issue of missing data for some pixels in few short periods, this study used the mean value of the reconstructed NDVI in the adjacent four years to fill these gaps.

### NOAA CDR NDVI

The NOAA CDR program provides a series of long-term, consistent, and accurate climate datasets used for monitoring and analyzing global climate change. The NOAA CDR daily NDVI is an important remote sensing indicator used to evaluate the status and changes of vegetation. The NOAA CDR daily NDVI maps with a spatial resolution of 0.05° by 0.05° used in this study were acquired from NOAA’s National Centers for Environmental Information, with a time span from 1981 to ten days before the present^20^. The fundamental data of this dataset includes data obtained by AVHRR sensor that equipped in eight NOAA polar orbiting satellites (NOAA-7, -9, -11, -14, -16, -17, -18, and -19) and VIIRS sensor that mounted on the SNPP and JPSS-1 (or NOAA-20) satellite platforms^20,21^. A series of ancillary data (e.g., digital elevation model, surface pressure, precipitable water, water vapor, and ozone) are also used in producing the NOAA CDR daily NDVI. This dataset has undergone systematic atmospheric correction, bidirectional reflectance distribution function (BRDF) correction, and cloud/cloud shadow identification. The reflectance spectra and NDVI constructed by the NOAA CDR program were compared and validated against MODIS data, demonstrating high levels of consistency (root mean square deviation, RMSD < 0.03 for red and NIR spectral bands, RMSD < 0.075 for NDVI) between the two datasets. In addition, a comparison with the daily Long Term Data Record (LTDR) observations at 48 global sites in 1999 also revealed the high reliability of this dataset. However, this original dataset only identified partial valid information and did not addressed the spatial gaps. Limitations such as data gaps and noise remain noticeable^20,21^.

### NDVI datasets for comparison

We validated the reconstructed results at different time scales. First, following a comparison with the original NOAA CDR valid NDVI, we further assessed the reliability of our reconstruction results at the daily scale by comparing with the MODIS MCD19A3CMG v6.1 daily continuous gap-filled NDVI^18^ between February 24, 2000 through May 10, 2023. Second, we conducted comparative validation at the monthly scale with three popular NDVI datasets—the third generation of Global Inventory Modeling and Mapping Studies (GIMMS3g) NDVI^34^, Systeme Probatoire d’Observation de la Terre (SPOT)/PROBA NDVI^35^, and MODIS MOD13C2 NDVI^36^. The GIMMS3g NDVI was constructed based on the daily NOAA AVHRR recordings by optimizing and integrating the original NDVI. This NDVI dataset has a temporal resolution of 15 days and a spatial resolution of 1/12° (approximately 8 km), spanning from 1982 to 2015^34^. The SPOT/PROBA NDVI merged vegetation datasets from SPOT/VEGETATION C3 and PROBA-V, and further adjusted the original data based on the bidirectional reflectance distribution function^37,38^. This dataset generated a global scale NDVI dataset over a long duration (from 1999 to 2020) with a spatial resolution of 1 km × 1 km and a temporal resolution of 10 days^35^. The MODIS NDVI dataset (MOD13C2) is obtained from the Terra satellite platform. The MOD13C2 NDVI maps were cloud-free spatial composites that retrieved from daily, atmosphere-corrected, bidirectional surface reflectance and generated at monthly intervals using maximum-value composite (MVC) method. The MOD13C2 NDVI data is projected on a 0.05-degree geographic climate modeling grid. This dataset has been improved by undergoing various calibration changes and showed high accuracy from various airborne and field validation campaigns^39^. We generated monthly NDVI of the aforementioned three datasets with a spatial resolution of 0.05° based on the MVC algorithm and nearest neighbor assignment method. The time span of the monthly GIMMS3g NDVI is from January 1982 to December 2015, and that of the monthly SPOT/PROBA NDVI is from January 1999 to September 2019. The monthly MOD13C2 NDVI spans from February 2000 to May 2023.

### Quality evaluation of the reconstructed NDVI

We adopted several approaches to validate the quality of the reconstructed daily NDVI. First, we quantified the accuracy of the reconstructed NDVI by comparing it with the original valid NOAA CDR NDVI at the pixel scale. Specifically, we calculated the *PB*, coefficient of determination (*R*^2^), and root mean square error (RMSE) of each pixel. We overlapped the above three maps to derive the RGB (Red-Green-Blue) combinations to visualize the spatial patterns of the quality of the reconstructed NDVI. Second, we analyzed the distribution of all pixels in nine geographical zones in a three-dimensional spatial coordinate system composed of |PB|, *R*^2^, and RMSE to evaluate the quality of the reconstructed NDVI in each zone. Third, we randomly selected three equidistantly distributed samples in each geographical zone (Supplementary Figure S2a), and quantified the matching degree of the temporal evolution characteristics between the reconstructed NDVI and the original valid NDVI at each sample point. In addition, we constructed a certain number of random points in each geographical zone (Supplementary Figure S2b), and evaluated the matching degree between the reconstructed NDVI and the original valid NDVI on all random points in each zone.

In addition to the aforementioned evaluation strategies, we conducted spatiotemporal consistency comparisons between the reconstructed NDVI and other NDVI datasets (i.e., MODIS MCD19A3CMG v6.1 daily continuous gap-filled NDVI, GIMMS3g monthly NDVI, SPOT/PROBA monthly NDVI, and MODIS MOD13C2 monthly NDVI). We evaluated the spatial patterns of the spatiotemporal consistency between the reconstructed NDVI and the comparative datasets by mapping the RGB combination of Pearson correlation coefficient (*r*), RMSE, and mean absolute error (MAE) between the reconstructed NDVI and the other NDVI datasets, respectively. We also analyzed the distribution patterns of these three statistical metrics along the latitude to systematically assess the differences between the reconstructed NDVI and the comparative datasets. The five criteria used in this study to evaluate the quality of the reconstructed NDVI include *r*, *R*^2^, MAE, RMSE, and *PB*.

1 $$r=\frac{{\sum }_{i=1}^{n}({Y}_{i,rec}-{\bar{Y}}_{rec})({Y}_{i,com}-{\bar{Y}}_{com})}{\sqrt{{\sum }_{i=1}^{n}{({Y}_{i,rec}-{\bar{Y}}_{rec})}^{2}}\sqrt{{\sum }_{i=1}^{n}{({Y}_{i,com}-{\bar{Y}}_{com})}^{2}}}$$

2 $${R}^{2}=1-\frac{{\sum }_{i=1}^{n}{({Y}_{i,obs}-{Y}_{i,rec})}^{2}}{{\sum }_{i=1}^{n}{({Y}_{i,obs}-{\bar{Y}}_{obs})}^{2}}$$

3 $${\rm{MAE}}=\frac{1}{n}\mathop{\sum }\limits_{i=1}^{n}\left|{Y}_{i,rec}-{Y}_{i,com}\right|$$

4 $${\rm{RMSE}}=\sqrt{\frac{1}{n}\mathop{\sum }\limits_{i=1}^{n}{\left({Y}_{i,rec}-{Y}_{i,com}\right)}^{2}}$$

5 $$PB=\frac{1}{n}\mathop{\sum }\limits_{i=1}^{n}\left(\frac{{Y}_{i,rec}-{Y}_{i,obs}}{{Y}_{i,obs}}\times 100 \% \right)$$

where *n* is the number of the samples. *Y*_*i,rec*_ and *Y*_*i,com*_ are the reconstructed NDVI and comparative data, respectively. ${\bar{Y}}_{rec}$ and ${\bar{Y}}_{com}$ are the mean values of reconstructed NDVI and comparative data, respectively. *Y*_*i,obs*_ and ${\bar{Y}}_{obs}$ are the original NOAA CDR valid NDVI and the corresponding mean value, respectively.

## Data Records

The daily gap-free NDVI dataset covers a spatial extent of 18.16°N to 53.56°N and 73.44°E to 135.09°E with a spatial resolution of 0.05°. The NDVI dataset from June 24, 1981 to May 10, 2023 is available at figshare repository^40^. The NDVI data for each year is individually stored as a standard Network Common Data Form, version 4 (NetCDF-4, NC4) format and a total of 43 NC4 files are provided. The file names follow the structure of “Daily_Gap-filled_NDVI_YYYY.nc4”, where “YYYY” represents the year. Additional information, such as the scale factor, is stored in the files.

## Technical Validation

### The reconstruction NDVI

**Fig. 2:**
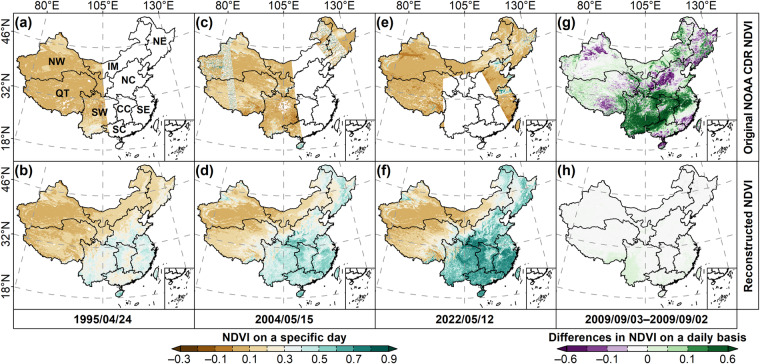
Visual comparison between the original daily NOAA CDR NDVI and the reconstructed daily NDVI maps. (**a**), (**c**), and (**e**) show the original NOAA CDR NDVI maps in three different dates, and (**b**), (**d**), and (**f**) present the reconstructed NDVI maps in the same dates, respectively. (**g**) shows the differences in the original NOAA CDR NDVI between September 3, 2009 and September 2, 2009, while (**h**) shows the difference in the reconstructed NDVI between these two days. NW, Northwest China; IM, Inner Mongolia; NE, Northeast China; QT, Qinghai-Tibetan Plateau; NC, North China; SW, Southwest China; CC, Central China; SE, Southeast China; SC, South China.

Figure 2a–f map the spatial distributions of the original NOAA CDR NDVI and the reconstructed NDVI in three specific days. We can see a large range of missing data in the original data. Our reconstructed NDVI maps filled these gaps in the original dataset. In addition, there are many unreliable data in the original dataset due to the impact of cloud and other noise. For example, Fig. 2g illustrates the difference map in the original dataset between September 3, 2009 and September 2, 2009. Large positive (>0.6) and negative (<−0.6) differences in NDVI within two days are widely distributed over China. It is certain that, in the real situation, there will be no significant difference in NDVI within a two-day period. We mapped the differences in the reconstructed NDVI within the same two-day period and there is no significant difference between the reconstructed NDVI within these two days (Fig. 2h). Overall, our reconstructed daily NDVI effectively filled the gaps in the original NOAA CDR NDVI and fixed the invalid data in the original dataset.

### Quantitative evaluation

#### Evaluation at random point scale

**Fig. 3:**
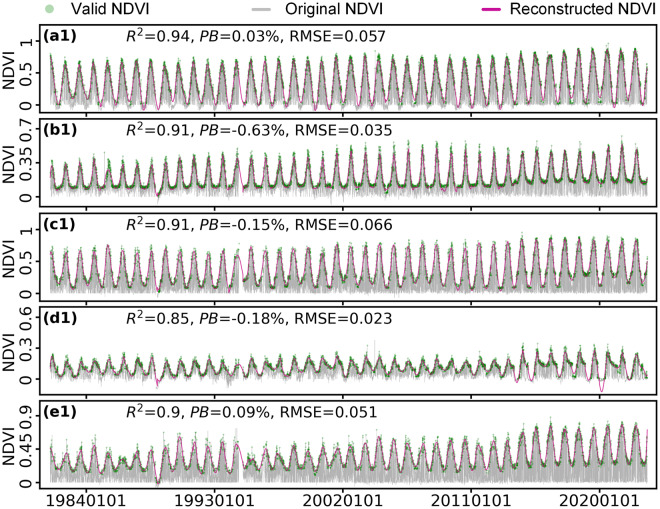
NDVI time-series analysis for the five random points. NDVI time-series of the other 22 sites were illustrated in Supplementary Figure S3 and Supplementary Figure S4. Serial numbers of the panels correspond to the random point label number in Supplementary Figure S2a.

We randomly selected three equidistantly distributed samples in each geographical zone (27 sites in total, Supplementary Figure S2a) and quantified the matching degree of the temporal evolution characteristics between the reconstructed NDVI and the original valid NOAA CDR NDVI at each site (Fig. 3, Supplementary Figure S3, and Supplementary Figure S4). The proportion of valid data in the entire time series showed an increasing trend from the southeastern China to the northwestern China due to more frequent impacts of cloud and rain in the humid and semi-humid regions in China. The distributions of daily NDVI showed substantial differences in different regions, even within the same geographical zone. Nevertheless, our reconstructed daily NDVI exhibited high consistency with the original valid NDVI across all sites (*R*^2^ ≥ 0.83, |PB| < 0.7%, and RMSE < 0.08), emphasizing the robustness of the reconstruction framework.

**Fig. 4:**
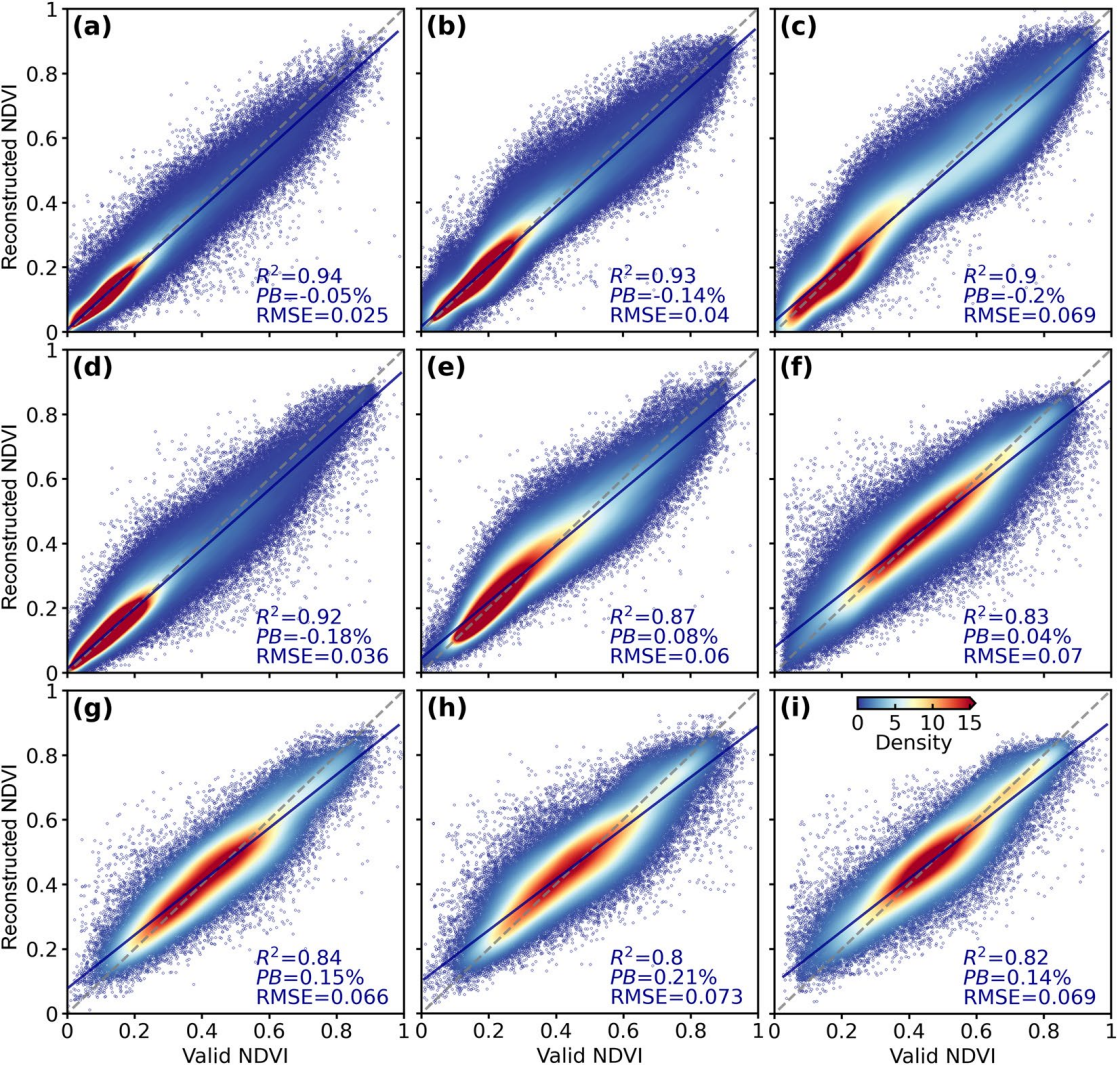
Comparison of the reconstructed daily NDVI with the original NOAA CDR valid NDVI at all random points (Supplementary Figure S2b) in the nine geographical zones. The gray dashed lines indicate the 1:1 lines and the blue solid lines are fitting lines. (**a**–**i**) represent Northwest China, Inner Mongolia, Northeast China, Qinghai-Tibetan Plateau, North China, Southwest China, Central China, Southeast China, and South China, respectively.

In addition, to fully evaluate the spatiotemporal reliability of our reconstructed daily NDVI, we drew a density scatter plot between the reconstructed NDVI and the original valid NDVI on a certain number of random sites in each geographical zone (Fig. 4). Scatter points in all plots are distributed nearby the 1:1 lines. High-density scatter points are tightly concentrated near the 1:1 lines. *R*^2^ between the reconstructed NDVI and original valid NDVI in the nine zones are all larger than 0.8, and the largest |PB| and RMSE are only 0.21% and 0.073, respectively. These evaluations confirmed the high spatiotemporal consistency between our reconstructed daily NDVI with the original valid NOAA CDR NDVI.

#### Evaluation at full pixel scale

**Fig. 5:**
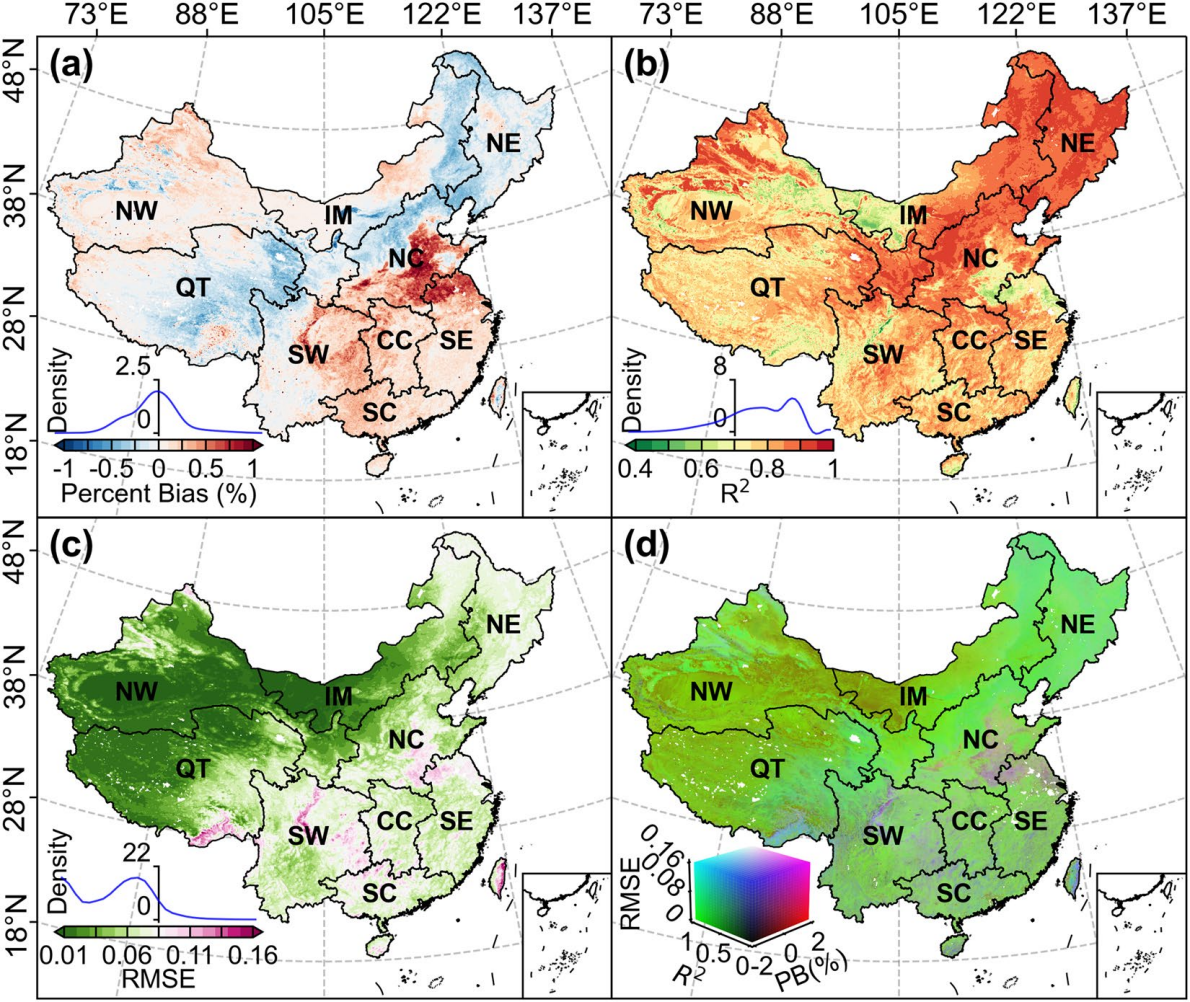
Spatial patterns of the spatiotemporal consistency between the reconstructed daily NDVI and the original NOAA CDR valid NDVI during the study period from 1981 to 2023. (**a**) *PB*, (**b**) *R*^2^, (**c**) RMSE, (d) RBG combination of the *PB*, *R*^2^, and RMSE maps. NW, Northwest China; IM, Inner Mongolia; NE, Northeast China; QT, Qinghai-Tibetan Plateau; NC, North China; SW, Southwest China; CC, Central China; SE, Southeast China; SC, South China.

Figure 5 shows the comparisons between the reconstructed daily NDVI and the original NOAA CDR valid NDVI. The *PB* between the two datasets over China are quite small with more than 99.91% of the study area showing a *PB* between −1% and 1% (Fig. 5a). The high positive *PB* (0.5%~1%) are mainly located in the border region between North China and Southeast China (accounting for an area ratio of 2.80%), where are mostly covered by farmlands. Negative *PB* are mainly distributed in Northeast China, Qinghai Tibetan Plateau, and the northwest of North China. However, most negative *PB* are larger than −0.5% with an area ratio of 48.41%. The overall average *PB* between the reconstructed NDVI and the original NOAA CDR valid NDVI is about −0.02%. *R*^2^ between the two datasets over China are ranging from 0.41 to 0.96 with high values in most parts of China, except for some farmlands in the North China Plain and deserts in the northwest of China (Fig. 5b). The overall average *R*^2^ between the two daily NDVI datasets over China is 0.79 with more than 96.22% of the study area showing an *R*^2^ larger than 0.6 and more than half of the study area (50.63%) owning an *R*^2^ larger than 0.8. The RMSE map of the two daily NDVI datasets shows that RMSE in more than 98.26% of the study area are smaller than 0.1 (Fig. 5c). The spatial pattern of high RMSE is similar to the *PB* map, which are mainly located in the border region between North China and Southeast China, some parts of Southwest China, and south of the Qinghai Tibetan Plateau. The overall mean value of RMSE in China is about 0.05. The above evaluation results indicate that our reconstructed daily NDVI are highly consistent with the NOAA CDR valid NDVI.

**Fig. 6:**
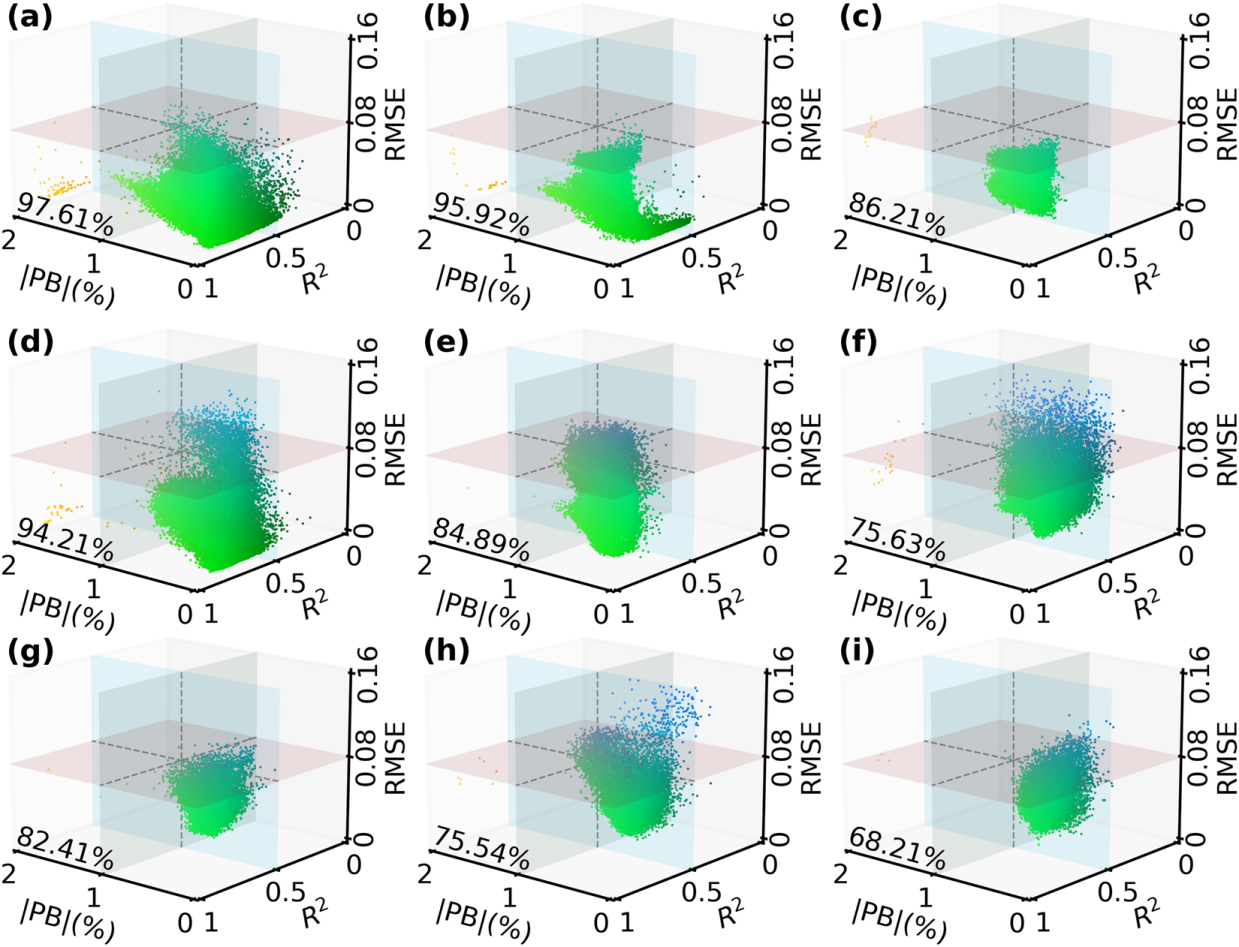
The pixel distributions of the nine geographical zones in a three-dimensional spatial coordinate system composed of absolute percent bias (|PB|), *R*^2^, and RMSE. (**a**–**i**) represent Northwest China, Inner Mongolia, Northeast China, Qinghai-Tibetan Plateau, North China, Southwest China, Central China, Southeast China, and South China, respectively. The percentage number in each panel indicates the proportion of pixels located in the quadrant with a |PB| < 1%, RMSE < 0.08, and *R*^2^ > 0.5. Colors of the scatter refer to the legend in panel (d) of Fig. 5.

We visualized the quality of the reconstructed NDVI over China by giving an RGB combination of the *PB*, *R*^2^, and RMSE maps (Fig. 5d). NDVI pixels with high quality should have low |PB|, low RMSE, but high *R*^2^. Here, we defined that a high-quality pixel should have a |PB| < 1%, RMSE < 0.08, and *R*^2^ > 0.5, simultaneously. Based on this definition, we found that the reconstructed NDVI in 89.76% of the study area showed high quality. These high-quality pixels are widely distributed throughout China, except for farmlands in the North China Plain and some hilly land in Southwest China and South China. The lower quality of these pixels is mainly due to the higher RMSE ( >0.08). We further analyzed the pixel distributions of the nine geographical zones in a three-dimensional spatial coordinate system composed of |PB|, *R*^2^, and RMSE (Fig. 6). It is clear that most pixels (>68%) in all zones are located in the quadrant with a |PB| < 1%, RMSE < 0.08, and *R*^2^ > 0.5 (i.e., high-quality pixel proportion). Northwest China, Inner Mongolia, and Qinghai-Tibetan Plateau present the largest high-quality pixel proportions (>94%) among all geographical zones. Overall, the high-quality pixel proportions of eight zones are larger than 75.5%, and only that in South China is relatively lower (68.21%). These pixel-based evaluations indicate the reconstructed NDVI in the nine geographical zones are reliable.

#### Comparison with MODIS daily gap-filled NDVI

**Fig. 7:**
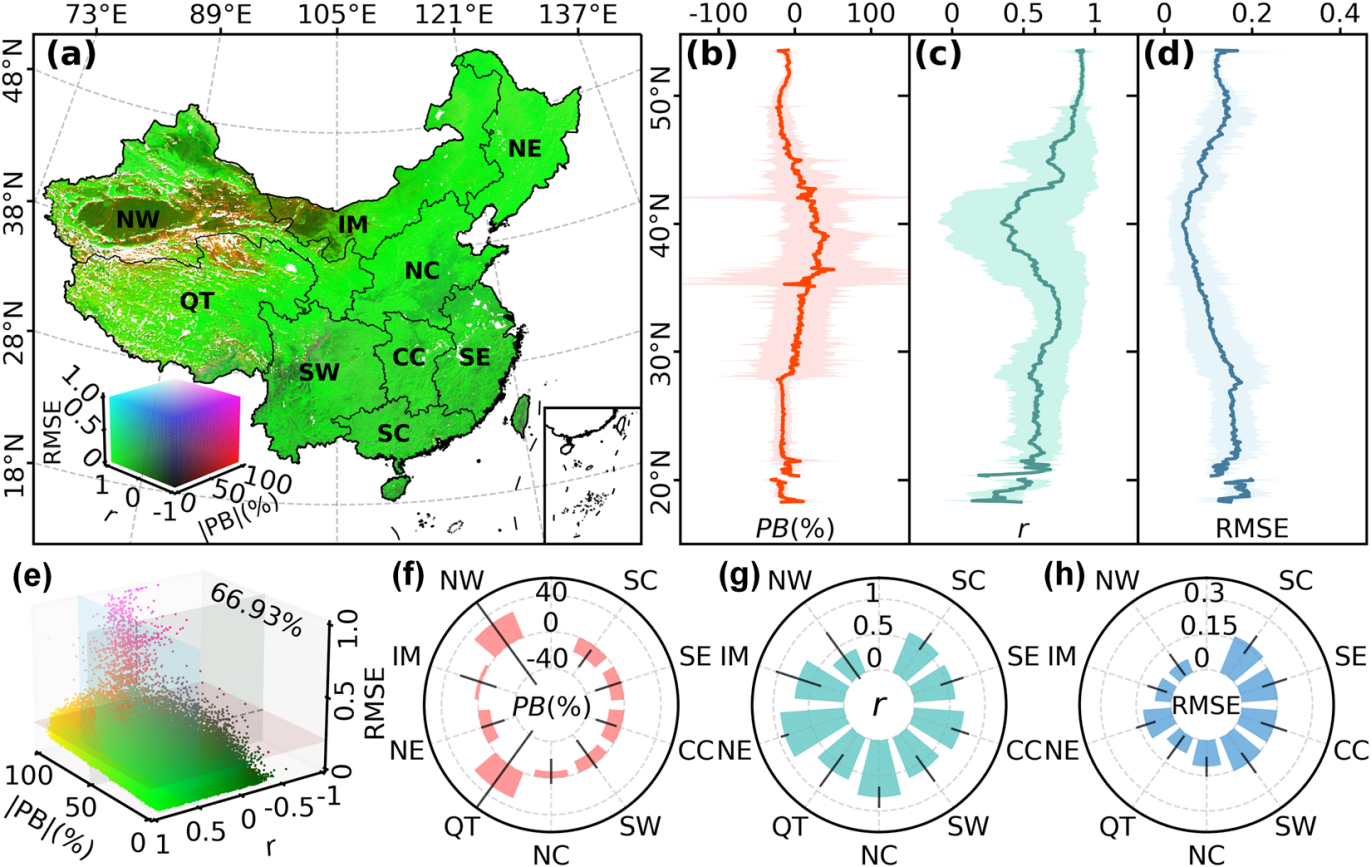
Comparison of the reconstructed daily NDVI with the MODIS gap-filled daily NDVI. (**a**) presents the RGB combination map of absolute percent bias (|PB|), *r*, and RMSE between the reconstructed NDVI and MODIS NDVI. (**b**–**d**) show the distributions of average *PB*, *r*, and RMSE along the latitude, respectively. (**e**) illustrates the pixel distributions in a three-dimensional spatial coordinate system composed of |PB|, *r*, and RMSE. The percentage number (66.93%) in panel (**e**) indicates the proportion of pixels located in the quadrant with a *r* > 0.5, |PB| < 50%, and RMSE < 0.2. Colors of the scatters refer to the legend in (**a**). (**f**–**h**) represent average *PB*, *r*, and RMSE between the reconstructed daily NDVI and the MODIS gap-filled daily NDVI in the nine geographical zones, respectively. Bars in (**f**–**h**) show the mean value of each criterion in the nine geographical zones with whiskers being the standard deviations. NW, Northwest China; IM, Inner Mongolia; NE, Northeast China; QT, Qinghai-Tibetan Plateau; NC, North China; SW, Southwest China; CC, Central China; SE, Southeast China; SC, South China.

Figure 7 shows the comparison of the reconstructed daily NDVI with the MODIS MCD19A3CMG v6.1 daily continuously gap-filled NDVI. The national average *r*, RMSE, and *PB* between the two daily NDVI datasets are 0.62, 0.1, and 7.54%, respectively. The RGB combination map of the *r*, RMSE, and |PB| maps (Fig. 7a) indicates that in 66.93% of the study area, our reconstructed NDVI shows relatively high spatiotemporal consistency (*r* > 0.5, |PB| < 50%, and RMSE < 0.2) with the MCD19A3CMG gap-filled NDVI (Fig. 7e). These regions are widely distributed throughout the country, except for the northwestern desert region and the southwestern mountainous area. The distribution of average *PB* along latitudes is shown in Fig. 7b. *PB* is relatively large between 28°N and 45°N with a substantial fluctuation range (i.e., standard deviation). The maximum *PB* occurs around 40°N, primarily due to differences between the two datasets in the northwestern desert regions, where vegetation coverages are quite sparse. In terms of the average *r* along latitudes (Fig. 7c), the average *r* exceed 0.5 for most latitudinal bands, except for regions around 40°N and 20°N. Similarly, the latitudinal average correlation coefficients corresponding to the northwestern desert regions exhibit larger fluctuations. The average RMSE at most latitudes are smaller than 0.2, with the lowest values observed around 40°N (Fig. 7d). This is mainly attributed to the relatively small NDVI values in the northwestern desert regions within this range. We quantified the average values of the three statistical criteria between our reconstructed NDVI and the MCD19A3CMG gap-filled NDVI in each geographical zone (Fig. 7f–h). The average |PB| in all zones, except for Northwest China and Qinghai-Tibetan Plateau, are smaller than 20%. The average *r* in all zones, except for Northwest China, are larger than 0.5. The largest regional average *r* (0.9) occurs in Northeast China. In terms of RMSE, the regional average RMSE in all zones are smaller than 0.17. The largest regional average RMSE (0.16) occurs in South China and the smallest regional average RMSE (0.06) occurs in Northwest China.

#### Comparison at monthly scale

**Fig. 8:**
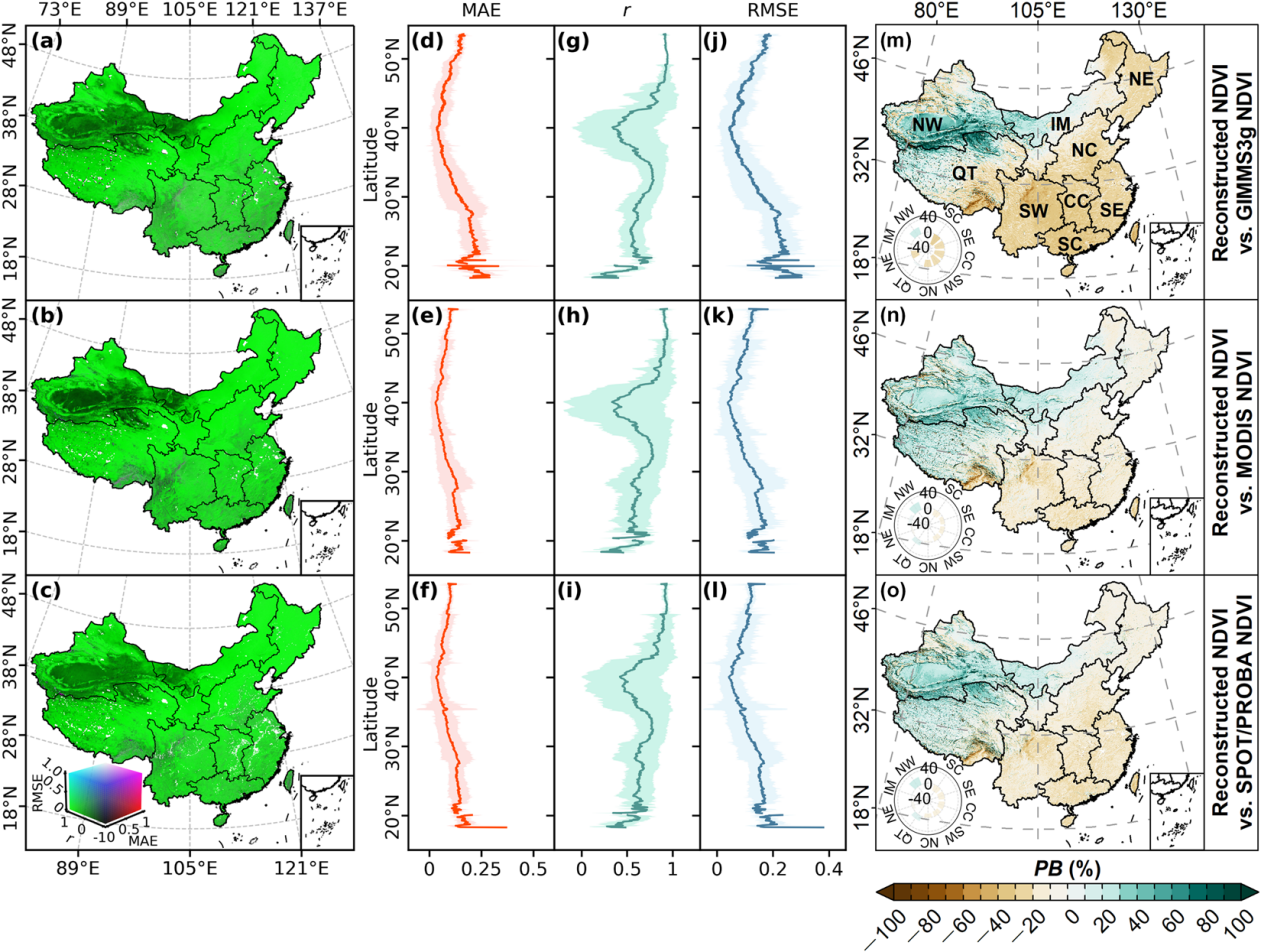
Comparison of the reconstructed NDVI with the other three NDVI datasets at the monthly scale. (**a**–**c**) present the RGB combination maps of MAE, *r*, and RMSE between the reconstructed NDVI and GIMMS3g NDVI (**a**), MODIS NDVI (**b**), and SPOT/PROBA NDVI (**c**), respectively. (**d**–**f**) show the distributions of average MAE along the latitude. (**g**–**i**) are the distributions of average *r* along the latitude. (**j**–**l**) present the distributions of average RMSE along the latitude. Shadow bands in (**d**–**l**) are the standard deviations along the latitude. (**m**–**o**) are percent bias (*PB*) maps between the reconstructed NDVI and GIMMS3g NDVI, MODIS NDVI, and SPOT/PROBA NDVI at the monthly scale, respectively. Left insets in (**m**–**o**) present the average *PB* of each geographical zone. NW, Northwest China; IM, Inner Mongolia; NE, Northeast China; QT, Qinghai-Tibetan Plateau; NC, North China; SW, Southwest China; CC, Central China; SE, Southeast China; SC, South China.

We further evaluated the reliability of our reconstructed NDVI by comparing with other three popular NDVI datasets (i.e., GIMMS3g NDVI, MODIS MOD13C2 NDVI, and SPOT/PROBA NDVI) on a monthly scale. Figure 8a–c map the RGB combinations of *r*, RMSE, and MAE between the monthly maximum NDVI of the reconstructed NDVI and the other three NDVI datasets, respectively. The spatial distributions of the RGB combinations of the three statistical criteria between our reconstructed NDVI and the three NDVI datasets present similar patterns over China. The reconstructed NDVI in most pixels ( > 75%) over China show a high significant positive correlation (*r* > 0.5, *p* < 0.05) with the other three datasets. Pixels with low *r* or negative *r* are mainly located in Southwest China, Qinghai Tibetan Plateau, and deserts in Northwest China. On the whole, the average *r* between our reconstructed NDVI and GIMMS3g NDVI, MODIS NDVI, and SPOT/PROBA NDVI are 0.65, 0.65, and 0.67, respectively. The corresponding average MAE (or RMSE) are 0.1, 0.07, and 0.08 (or 0.12, 0.09, and 0.1), respectively.

Figure 8d–l present the distributions of average MAE, *r*, and RMSE along the latitude, respectively. The average MAE and RMSE at most latitudes are smaller than 0.25 and show the lowest values at about 40°N in the three comparisons, presenting an increasing pattern with the decrease of latitude (Fig. 8d–f, Fig. 8j–l). In contrast, the latitudinal average *r* between our dataset and other three datasets all show a decreasing trend with the decrease of latitude (Fig. 8g–i). The average *r* at most latitudes in the three comparisons are larger than 0.5. However, there is a noticeable low-value region with high fluctuation range in the distribution curves of *r*, primarily attributed to the low correlations between our dataset and the three comparative datasets in the desert region of Northwest China. The *PB* maps between monthly maximum NDVI of our dataset and the three comparative datasets are shown in Fig. 8m–o. The spatial distributions of the three *PB* maps are similar, showing an increasing gradient from southeast to northwest. The national average *PB* between our dataset and GIMMS3g NDVI, MODIS NDVI, and SPOT/PROBA NDVI are −5.79%, 4.82%, and 2.66%, respectively.

## Usage Notes

In this study, we established a daily NDVI reconstruction framework by integrating valid data identification method with spatiotemporal sequence gap-filling technique and provided the first long-term (1981~2023) gap-free daily NDVI dataset over China with a spatial resolution of 0.05°. We adopted several approaches to validate the reliability of the reconstruction framework and the accuracy of the reconstruction NDVI. Our systematic evaluation indicated that the framework effectively addressed the inefficiency encountered by previous reconstruction methods when dealing with extensive data gaps and successfully filled these gaps and restored contaminated data in the original dataset.

The reconstructed daily NDVI dataset can provide crucial data support for better understanding the vegetation responses under climate and environmental changes, especially for rapidly occurring climate extremes^41–43^. Furthermore, our reconstructed daily gap-free NDVI can offer essential data support for improving the inversion reliability and temporal resolution of related vegetation parameters (e.g., gross primary productivity and net primary productivity^44^). For instance, the conventional satellite-based light use efficiency models (e.g., the Carnegie-Ames-Stanford Approach, CASA) use monthly maximum NDVI to simulate the fraction of photosynthetically active radiation (FPAR), which is subsequently used for the estimates of monthly vegetation productivity^45–47^. Vegetation productivity models based on monthly maximum vegetation indices may overestimate monthly vegetation productivity. Our reconstructed daily gap-free NDVI dataset can help improve the temporal resolution of these model estimates while also enhance the reliability of the simulation results.

All the daily NDVI data are stored in the NC4 format at the figshare repository. Users can use Python, R, and MATLAB platforms to read and manipulate the data. It should be noted that the data must be multiplied by the scale factor (0.001).

### Supplementary information

Supplementary Information

## Data Availability

The Python codes for generating and processing the daily gap-free NDVI data in China can be accessed through GitHub (https://github.com/mainearth/Daily-Gap-free-NDVI-Code.git).
