# Supplementary material for: A daily gap-free normalized difference vegetation index dataset from 1981 to 2023 in China
Source: Sci Data. 2024 May 22;11:527. doi: 10.1038/s41597-024-03364-3 (PMC11111700; doi:10.1038/s41597-024-03364-3)
Supplement: Supplementary file 1 — Supplementary Information [file 41597_2024_3364_MOESM1_ESM.pdf]

Supplementary Information for

A daily gap-free normalized difference vegetation index dataset from 1981 to 2023 in China

Authors

Huiwen Li[1,2], Yue Cao[3], Jingfeng Xiao[4], Zuoqiang Yuan[1,*], Zhanqing Hao[1], Xiaoyong Bai[5,#], Yiping Wu[6], Yu Liu[1]

Affiliations

1. School of Ecology and Environment, Northwestern Polytechnical University, Xi'an, Shaanxi Province 710129, China
2. Technology Innovation Center for Natural Ecosystem Carbon Sink, Ministry of Natural Resources, Kunming, Yunnan Province 650111, China
3. Xi'an Institute for Innovative Earth Environment Research, Xi'an, Shaanxi Province 710061, China
4. Earth Systems Research Center, Institute for the Study of Earth, Oceans, and Space, University of New Hampshire, Durham, NH 03824, USA
5. State Key Laboratory of Environmental Geochemistry, Institute of Geochemistry, Chinese Academy of Sciences, Guiyang, Guizhou Province 550081, China
6. Department of Earth & Environmental Science, Xi'an Jiaotong University, Xi'an, Shaanxi Province 710049, China

corresponding authors: Zuoqiang Yuan (zqyuan@nwpu.edu.cn); Xiaoyong Bai (baixiaoyong@vip.skleg.cn)

Table of contents

**Supplementary Figures**		
Figure S1	Annual average absolute percent bias (|PB|) between monthly $NDVI_{max}$ and previous adjacent month (a) and annual maximum |PB| between monthly $NDVI_{max}$ and the average $NDVI_{max}$ of the same month in adjacent four years (b).	2
Figure S2	Spatial distributions of the random points for the quality evaluation of the reconstructed daily NDVI in the study area.	3
Figure S3	NDVI time-series analysis for the 11 random points.	4
Figure S4	NDVI time-series analysis for the other 11 random points.	5

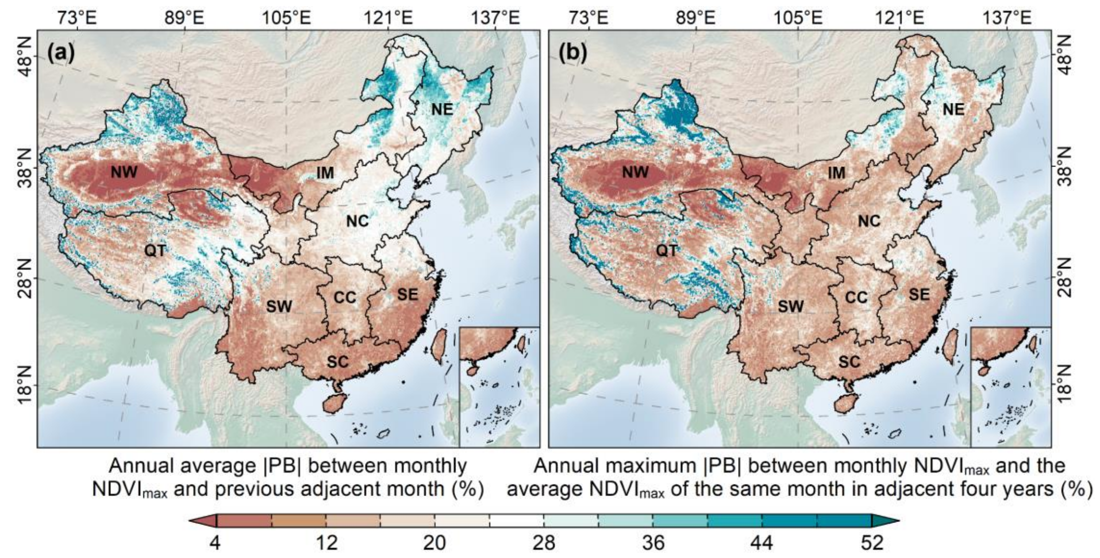

Supplementary Figure S1 Annual average absolute percent bias (|PB|) between monthly $NDVI_{max}$ and previous adjacent month (a) and annual maximum |PB| between monthly $NDVI_{max}$ and the average $NDVI_{max}$ of the same month in adjacent four years (b). The national average value of the map in panel (a) is about 20.39%. The national average value of the map in panel (b) is about 39.26%. NW, Northwest China; IM, Inner Mongolia; NE, Northeast China; QT, Qinghai-Tibetan Plateau; NC, North China; SW, Southwest China; CC, Central China; SE, Southeast China; SC, South China.

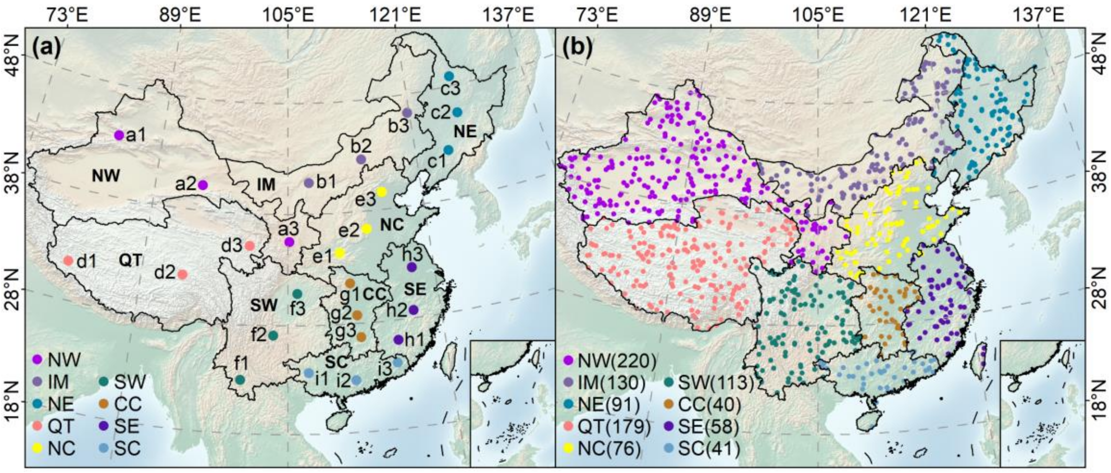

Supplementary Figure S2 Spatial distributions of the random points for the quality evaluation of the reconstructed daily NDVI in the study area. (a) maps the random points that equidistantly distributed in the nine geographical zones in China for the validation of the temporal variation of daily NDVI. There are three random points in each zone. (b) shows the random points for the validation of the spatiotemporal variation of daily NDVI. Numbers in the legend of panel (b) indicate the number of random points in each zone. The boundaries of the nine geographical zones in China are collected from (Li et al. 2022a). NW, Northwest China; IM, Inner Mongolia; NE, Northeast China; QT, Qinghai-Tibetan Plateau; NC, North China; SW, Southwest China; CC, Central China; SE, Southeast China; SC, South China.

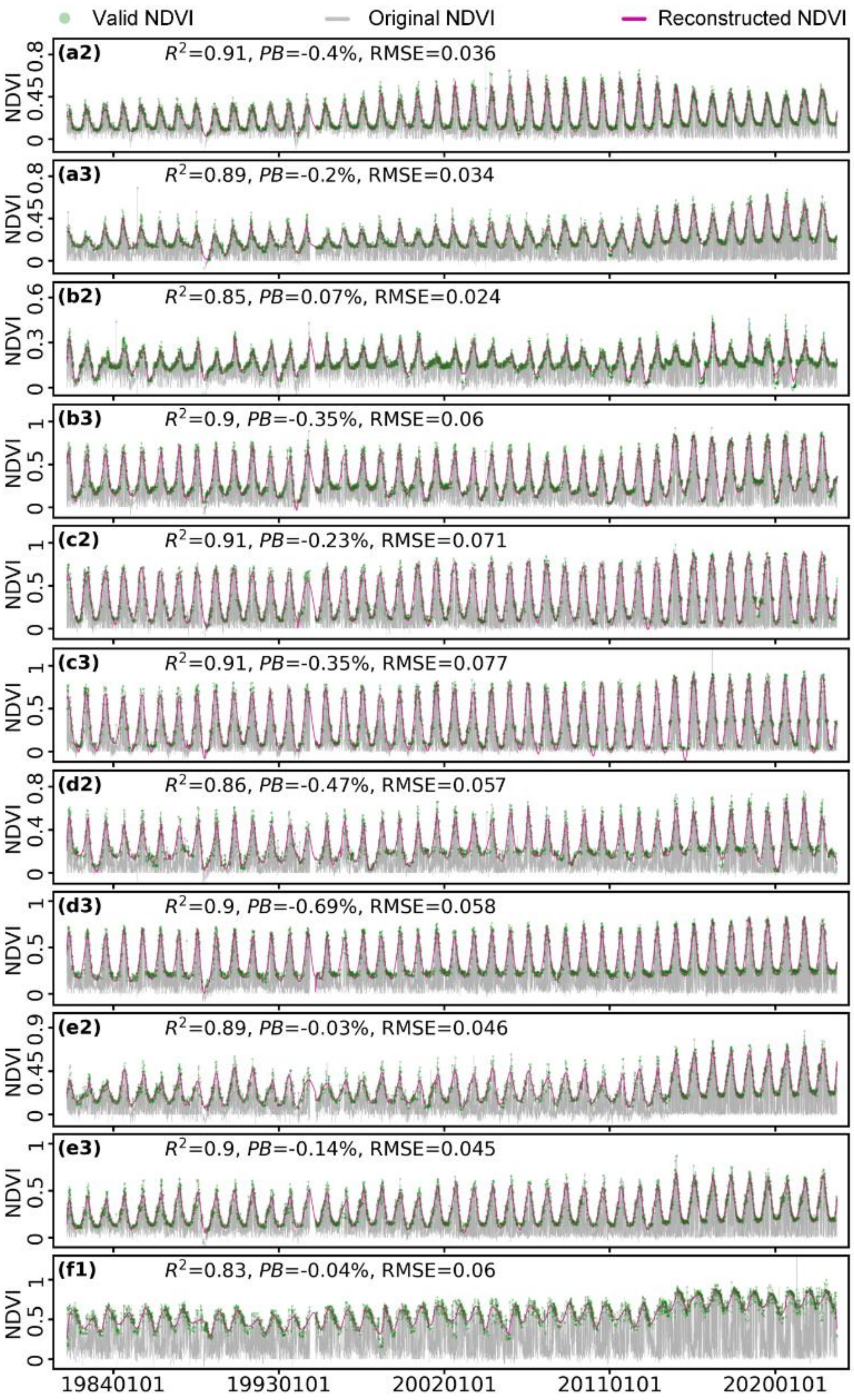

Supplementary Figure S3 NDVI time-series analysis for the 11 random points. Serial numbers of the panels correspond to the random point label number in **Supplementary Figure S2a**.

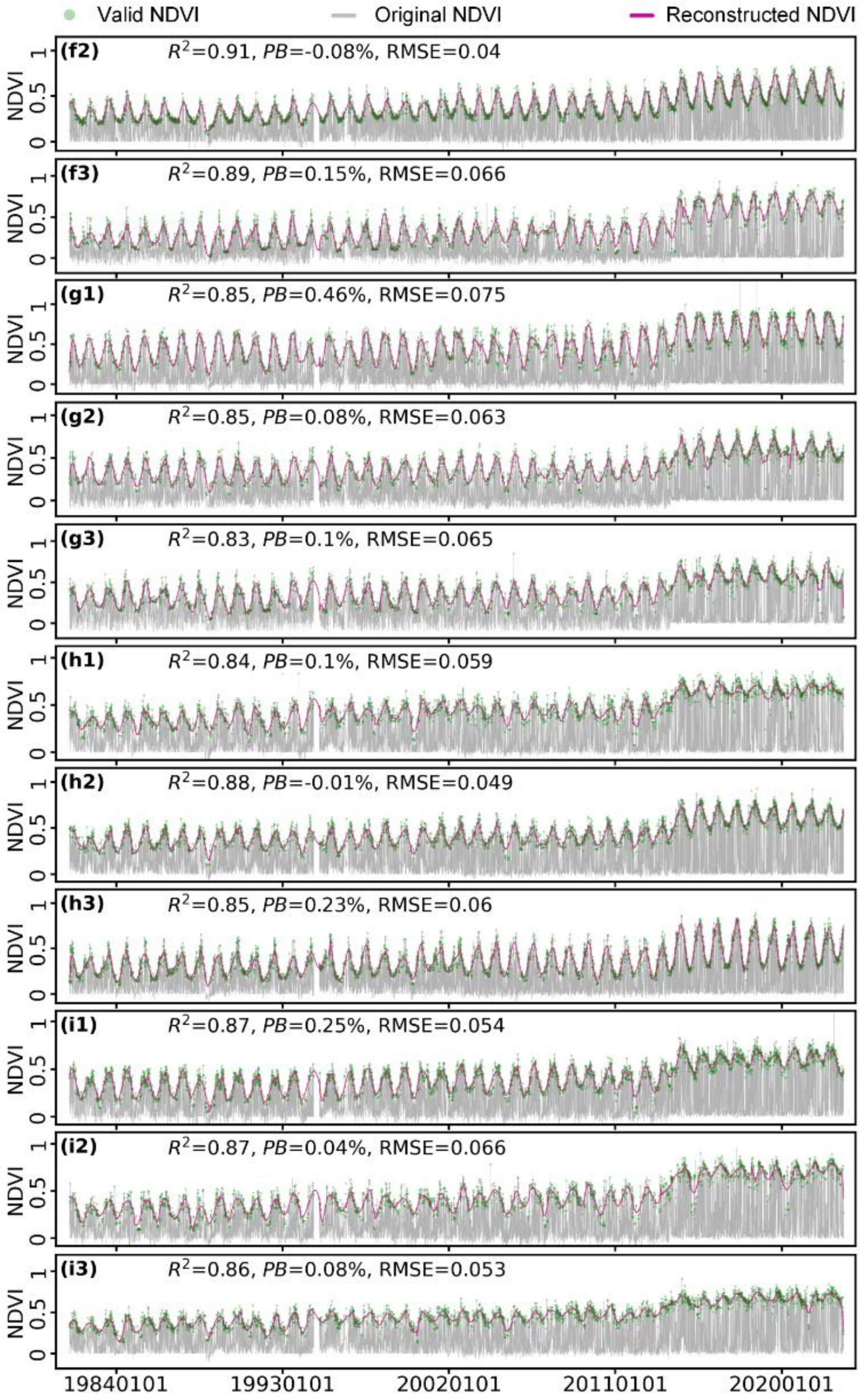

Supplementary Figure S4 NDVI time-series analysis for the other 11 random points. Serial numbers of the panels correspond to the random point label number in **Supplementary Figure S2a**.
